# *SLC22A3* methylation-mediated gene silencing predicts adverse prognosis in acute myeloid leukemia

**DOI:** 10.1186/s13148-022-01373-w

**Published:** 2022-12-02

**Authors:** Yu Gu, Zi-jun Xu, Jing-dong Zhou, Xiang-mei Wen, Ye Jin, Qian Yuan, Pei-hui Xia, Yuan Feng, Lei Yang, Jiang Lin, Jun Qian

**Affiliations:** 1 Department of Hematology, Affiliated People’s Hospital of Jiangsu University, 8 Dianli Rd., Zhenjiang, 212002 Jiangsu People’s Republic of China; 2 Zhenjiang Clinical Research Center of Hematology, 8 Dianli Rd., Zhenjiang, 212002 Jiangsu People’s Republic of China; 3 Laboratory Center, Affiliated People’s Hospital of Jiangsu University, Zhenjiang, Jiangsu People’s Republic of China; 4 The Key Lab of Precision Diagnosis and Treatment in Hematologic Malignancies of Zhenjiang City, Zhenjiang, Jiangsu People’s Republic of China

**Keywords:** Acute myeloid leukemia, Methylation, *SLC22A3*, 5-aza-2′-deoxycytidine

## Abstract

**Background:**

We screened out several hypermethylated solute carrier (SLC) family genes in acute myeloid leukemia by reduced representation bisulfite sequencing. *SLC22A3* encodes an organic cation transport protein, which is critical for drug transportation and cellular detoxification. *SLC22A3* is significantly downregulated and associated with tumor progression and worse prognosis in a variety of solid tumors. However, there are no data available regarding the role of *SLC22* in AML. This study aimed to explore the regulatory mechanism of DNA methylation on *SLC22A3* expression, as well as its clinical significance in AML prognosis.

**Results:**

*SLC22A3* was identified as the sole prognosis-associated gene among *SLCs* based on TCGA and Beat AML databases. Bone marrow mononuclear cells (BMMNCs) from AML, MDS patients, and healthy donors were enrolled in this study. *SLC22A3* methylation was significantly increased in AML compared with controls and MDS patients; meanwhile, the expression level of *SLC22A3* was decreased. *SLC22A3* hypermethylation presented an obvious association with some specific clinical characteristics and affected the survival time of AML patients as an independent risk indicator. *SLC22A3* expression changed regularly as the disease complete remissions and relapses. Demethylation drug 5-aza-2′-deoxycytidine (DAC) activated transcription and increased mRNA expression of *SLC22A3* in leukemia cell lines and AML fresh BMMNCs. Knockdown of *SLC22A3* in leukemia cells enhanced cell proliferation and suppressed cell apoptosis. Data from public programs were used for auxiliary screening of probable molecular mechanisms of *SLC22A3* in the antileukemia effect.

**Conclusions:**

Our results showed that increased methylation and decreased expression of *SLC22A3* may be indicators of poor prognosis in AML. Methylation-silenced *SLC22A3* expression may have potential guiding significance on antileukemia effect of DAC.

**Supplementary Information:**

The online version contains supplementary material available at 10.1186/s13148-022-01373-w.

## Introduction

Recent decades have witnessed an increasing trend of leukemia incidence and mortality in china and worldwide [1–3]. AML is the most common adult acute leukemia that varies greatly in clinical features, immune phenotypes, morphology and genetics. Except for all-trans-retinoic acid (ATRA) for the treatment of acute promyelocytic leukemia, AML with conventional induction consolidation chemotherapy alone has a poor prognosis, low long-term survival rate and high recurrence rate. Hence, there is a high medical need to improve the outcome of AML patients [4–6]. Myelodysplastic syndrome (MDS) is a heterogeneous clonal myeloid neoplasm that is characterized by ineffective hematopoiesis and cytopenia in one or more of the myeloid lineages, as well as an apparent risk of progression to AML [7].

AML is commonly associated with a variety of different genetic abnormalities, such as chromosomal abnormalities, heterozygous deletions, gene mutations, and epigenetic abnormalities [8]. CpG island methylation is usually altered during malignant transformation, which plays an important role in transcriptional regulation and offers new ideas for AML and MDS surveillance and treatment [9]. SLCs are the largest family of transmembrane transport proteins. Dysregulation and mutations of SLC encoding gene have been associated with susceptibility to a variety of diseases, including metabolic disorders and many kinds of cancers [10–12]. The solute carrier family 22 (SLC22) members mainly contain the organic cation transporters OCT 1 (SLC22A1), OCT2 (SLC22A2), and OCT3 (SLC22A3), which are involved in many metabolic and detoxification processes as transcription factors. SLC22A3 has the widest tissue distribution among SLC22s [13]. Early studies focusing on metabolic functions and metabolic diseases found that SLC22A3 is critical for eliminating endogenous small organic cations and drugs [14, 15]. To date, many research works have indicated the association between *SLC22A3* and cancers, including tumorigenesis, tumor invasion and metastasis, uptake and metabolism of antineoplastic drugs, and disease prognosis [16–22], but the significance of *SLC22A3* in AML remains unclear so far. Therefore, we studied *SLC22A3*, one of several hypermethylated genes that we screened for in AML. Our aim was to investigate whether DNA methylation and mRNA expression of *SLC22A3* are associated with AML and have potential value as a drug target for AML treatment.

## Materials and method

### Patients and samples

Three hundred and thirty-six adult AML patients in various clinical statuses [including 271 newly diagnosed cases, 66 complete remission (CR) cases, and 24 relapsed cases], 93 newly diagnosed MDS patients, and 45 healthy donors were enrolled in this study. BMMNCs were isolated by Ficoll density gradient centrifugation. Our research was approved by the Institutional Ethics Committee of the Affiliated People’s Hospital of Jiangsu University, and each individual provided signed informed consents for their participation. The diagnosis and classification of MDS and AML patients were based on the French–American–British (FAB) and the 2016 World Health Organization (WHO) criteria [7, 23].

### Cytogenetic and molecular genetic analysis

A series of clinic-hematological profiles and auxiliary examination results of patients were enrolled in this study for clinical correlation research. Hematologic laboratory results, including morphologic identification of bone marrow aspirate, cytogenetic analysis, immune-phenotypic feature, and molecular testing were valuable for the diagnosis and prognosis of hematopoietic malignancies. These indexes were also applied to follow-up study after induction and consolidation chemotherapy [24, 25]. Cytogenetic characteristics were analyzed by R-banded standard karyotyping and/or fluorescence in situ hybridization at diagnosis. Twelve commonly gene mutations tested by high-resolution melting analysis and/or direct DNA sequencing were carried out on BMMNCs [4]. The prognosis of MDS patients can be grouped into four categories using the International Prognostic Scoring System (IPSS): low risk, INT‐1, INT‐2, and high risk [26, 27].

### RNA isolation and real-time quantitative polymerase chain reaction (RT-qPCR)

Total RNA was isolated as per the TRIzol reagent instruction (Invitrogen, Carlsbad, CA, USA) from each BMMNCs sample and was transcriptionally reversed into cDNA using PrimeScript™ II 1st Strand cDNA Synthesis Kit (TaKaRa, Tokyo, Japan). RNA concentration and quality were assessed with a NanoDrop 2000 (NanoDrop Technologies, Wilmington, Delaware USA). *SLC22A3* expression was detected by RT-qPCR using the SYBR Premix Ex Taq II (TaKaRa, Tokyo, Japan) and was calculated by relative expression level (2 − ΔΔCt) with ABL as internal reference [25]. Primers sequence: *SLC22A3* (71 bp), 5′-CCACCATCGTCAGCGAGT-3′ (forward), 5′-CAGGATGGCTTGGGTGAG-3′ (reverse); internal reference *ABL* (118 bp), 5′-TCCTCCAGCTGTTATCTGGAAGA-3′ (forward), 5′-TCCAACGAGCGGCTTCAC-3′ (reverse). The appropriate primers and personalized RT-qPCR reaction temperature partly different from the manufacturer’s template were verified by gel electrophoresis and Sanger sequencing of PCR products, 60 °C for 30 s in annealing temperature and 82 °C for 30 s in collected fluorescence temperature.

### DNA isolation and bisulfate modification

Genomic DNA was isolated from pretreated BMMNCs samples by Genomic DNA Purification Kit (Gentra, Minneapolis, MN, USA) and then modified using CpGenome DNA Modification Kit (Chemicon, Temecula, Canada). Methylation sites in genomic DNA were exposed after the bisulfite conversion and can be identified by targeted primers [28].

### Targeted bisulfite sequencing assay and real-time quantitative methylation-specific PCR (RQ-MSP)

Based on the CpG sites of the genomic promoter region, we designed the primers and check them for feasibility and specificity. The bisulfite convert ratio and methylation levels of 27 CpG sites located at *SLC22A3* promoter were validated by targeted bisulfite sequencing (methyl target) sequencing (Genesky Biotechnologies Inc., Shanghai, China), a multiple targeted CpG methylation analysis by next-generation sequencing [29]. The 20μL RQ-MSP reaction system was operated with 20 ng modified DNA, 0.8 μM primers, 10 μM SYBR Premix Ex Taq II Mix and 0.4 μM ROX Reference Dye II (TaKaRa, Tokyo, Japan). Primers sequence: methylated *SLC22A3* (M-*SLC22A3*, 252 bp), 5′-GGGATTAAAAGGAGTTTCGC -3′ (forward), 5′-CACTCGCCCTAACGCTATAC -3′ (reverse); unmethylated *SLC22A3* (U-*SLC22A3*, 252 bp), 5′-GTAGGGATTAAAAGGAGTTTTGT -3′ (forward), 5′-CCTCACTCACCCTAACACTATAC -3′ (reverse); internal reference *ALU* (110 bp), 5′-TTAGGTATAGTGGTTTATATTTGTAATTTTAGTA-3′ (forward), 5′-ATTAACTAAACTAATCTTAAACTCCTAACCTCA-3′ (reverse). The relative methylation level of *SLC22A3* was calculated by the formula: N_*M/U-SLC22A3*_ = 2^ΔCT *M/U−SLC22A3*(control−sample)^ ÷ 2^ΔCT *ALU*(control−sample)^(2^−ΔΔCT^). RQ-MSP reaction systems were personalized in annealing and collected fluorescence temperatures, 66 °C (M) or 63 °C (U) for 30 s and 80 °C for 30 s, respectively.

### Human leukemia cell lines and AML fresh bone marrow mononuclear cells culture

The human leukemia cell lines K562 (FAB-M6), HL-60 (FAB-M2), THP-1 (FAB-M5), U937 (FAB-M5), SKM-1 (MDS-derived AML M5), and MV4-11 (FAB-M5) were purchased from the American Type Culture Collection (ATCC, Manassas, VA, USA) and gifted from other laboratories. Cells were cultured in freshly prepared RPMI 1640 medium (WISENT, Nanjing, China) supplemented with 10% fetal calf serum (ExCell Bio, Shanghai, China) and 1% penicillin–streptomycin (Hyclone, Shanghai, China), surrounded by a 37^◦^C humidified atmosphere containing 5% CO_2_.

Fresh mononuclear cells from the bone marrow of four newly diagnosed untreated AML patients (P1-M2a, P2-M2b, P3-M4a, and P4-M5b) were separated through density gradient centrifugation using a PBMC separation tube (FcMACS, NanJing, China) and suspended in Dulbecco's phosphate-buffered saline (WISENT, Nanjing, China) for twice, according to the specification protocol. After cultured in the same medium and environment as cell lines for 48 h, all the suspension cells were collected for further culture, while mesenchymal stem cells which grew by static adherence were isolated.

### RNA interference (RNAi)

Gene silencing induced by small interfering RNAs (siRNAs) was performed in HL60, K562, and AML fresh BMMNCs. siRNAs against *SLC22A3* (siSLC22A3) and its related negative control (siNC) were designed and synthesized by GenePharma (Shanghai, China). Cell transfection was performed using EntransterTM-R4000 (Engreen Biosystem, Beijing, China).

### Cell proliferation and apoptosis analysis

Cell lines (1.5 × 10^5^ cells/ml) and AML fresh BMMNCs (1 × 10^5^ cells/ml) were seeded onto a 6-well plate with normal substrate environment. After culturing for 0, 24, 48, and 72 h, cell proliferation status was counted on the counting board three times.

Cells (2 × 10^5^ cells/ml) were seeded onto a 6-well plate in RPMI 1640 medium without fetal calf serum for 72 h. Cell apoptosis rate was detected by flow cytometry (Beckman Coulter, Miami, FL, USA) using Annexin V-PE/7-AAD apoptosis detection kits (BD Pharmingen, San Diego, CA, USA). Viable cells were counted using 0.4% trypan blue staining (Biosharp, Anhui, China). Each experiment was repeated three times.

### Demethylation drug DAC sensitivity study

According to *SLC22A3* expression and methylation pattern in above six leukemic cell lines, we selected HL60 and K562 cells for further 5-Aza-2′-deoxycytidine (DAC, Sigma-Aldrich, St. Louis, MO, USA) sensitivity experiment. Normal growing cells lines and AML fresh BMMNCs (1 × 10^5^ cells/ml) were treated by DAC at a final concentration of 0 μM, 0.1 μM, 1 μM, and 10 μM for 72 h, respectively. The expression of *SLC22A3* in treated cells at each concentration was determined by RT-qPCR. After treated by DAC at a final concentration of 1 μM for 24 h, cell apoptosis of K562 siNC/siSLC22A3 was detected by flow cytometry.

### Sequencing and bioinformatics analyses

Our laboratory detected many differential methylation genes within four normal donors and four pairs of MDS-sAML (secondary AML) patients through RRBS [30]. The raw data have been submitted to NCBI SRA databases, whose accession number was PRJNA670308.

Gene expression (RNA Seq V2 RSEM), methylation (HM450), and clinical information of 200 adults with de novo AML (NEJM 2013) from the Cancer Genome Atlas (TCGA) dataset were downloaded via cBioPortal (http://www.cbioportal.org) and DiseaseMeth version 2.0 (http://bio-bigdata.hrbmu.edu.cn/diseasemeth/index.html) [31]. Genome-wide DNA methylation profiling of 15 AML and 5 normal bone marrow specimens from GSE63409 (http://www.ncbi.nlm.nih.gov/geo/; GSE63409) were used for gene screening. Genomic sequencing and clinical data of 558 cases from Beat AML program were downloaded via cBioPortal (http://www.cbioportal.org) [32]. Differential gene expression and enrichment analyses were calculated as reported previously [33].

### Statistical analyses

SPSS 22.0 software package and GraphPad Prism 5 were applied to statistical analyses. Student's t test (Mann–Whitney’s *U* test and Paired *T* test) were performed to compare the differences of continuous variables. The Pearson chi-square analysis/Fisher exact test was conducted to analyze the diversities of categorical variables, while Spearman correlation test was used to evaluate the correlation between genes expression and methylation. The ROC curve and area under the ROC curve (AUC) were used for assessing discriminative capacity of *SLC22A3* methylation between patients and controls. Kaplan–Meier and Cox regression (univariate and multivariate) analyses were carried out to evaluate the impact of *SLC22A3* on survival. Statistical significance was set at *P* < 0.05, and each test was two-sided.

## Results

### Screening of methylation-related candidate SLCs involved in AML prognosis by public database

**Fig. 1:**
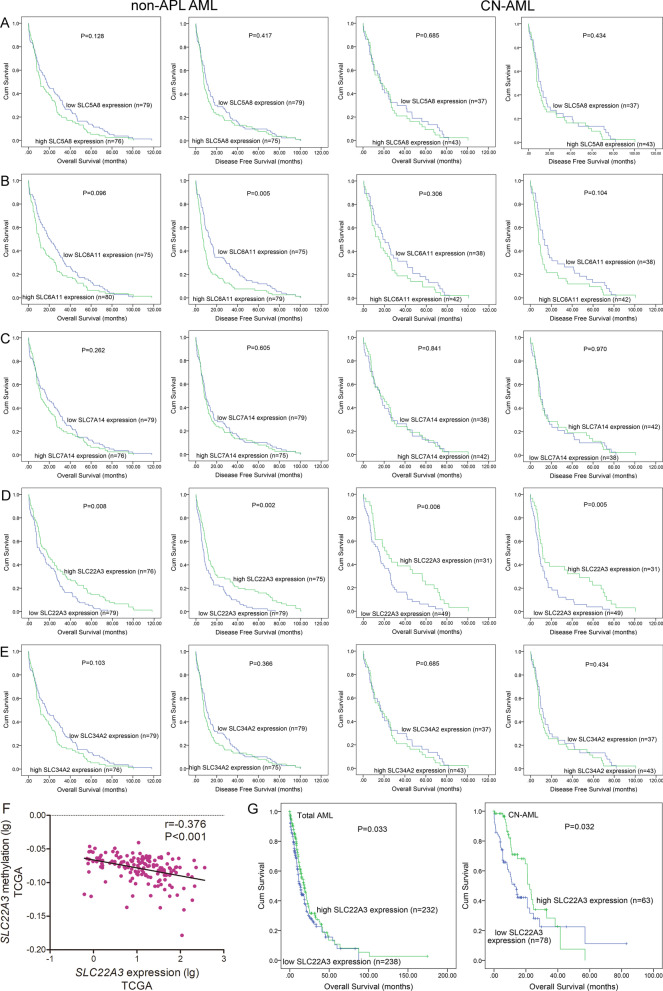
Identification of SLCs expression associated with prognosis in AML by public database. **A**–**E** *SLC5A8*, *SLC6A11*, *SLC7A14*, *SLC22A3*, *SLC34A2*. The impact of SLCs expression on OS and DFS was detected among non-APL AML and CN-AML patients from TCGA databases. AML patients were divided into two groups by the median mRNA expression level of each gene, respectively. **F** Correlation between DNA methylation and mRNA expression of *SLC22A3* in AML from TCGA database. **G** The impact of *SLC22A3* expression on OS was detected among total-cohort AML and CN-AML patients from beat AML database. AML patients were divided into two groups by the median *SLC22A3* expression level

Combined with our previous genome-wide methylation pattern research by RRBS [30] and the public methylation array GSE63409 data, we identified numerous abnormally hypermethylated genes including a group of *SLC* gene family members in AML, which have arose our attention (Additional file 1). Then, we screened genes that associated with AML prognosis among these *SLCs* based on TCGA database (Fig. 1A–E). Prognostic significance of each gene was evaluated between two groups divided by the median level of their expression. Kaplan–Meier analysis showed that high expression of *SLC22A3* predicted longer overall survival (OS) and disease-free survival (DFS) in both non-acute promyelocytic leukemia (APL) AML (non-APL AML) (*P* = 0.008 and 0.002) and cytogenetically normal AML (CN-AML) patients (*P* = 0.006 and 0.005; Fig. 1E). Independent assessments of paired *SLCs* methylation and expression sequencing data from TCGA AML project revealed a negative correlation between those of *SLC22A3* (*r* =  − 0.376, *P* < 0.001, *n* = 155, Fig. 1F), but not of other four genes (*SLC5A8*, *SLC6A11*, *SLC7A14*, and *SLC34A2*) (Additional file 2). Moreover, Kaplan–Meier analysis based on Beat AML patients also revealed this clinical significance between *SLC22A3* high-expression and longer OS in both whole-cohort AML and CN-AML (*P* = 0.033 and 0.032; Fig. 1G). We also observed SLC22A3 up-regulation in MLLT3-KMT2A rearranged AML and FLT3-ITD negative mutations according to Beat AML cohort (Additional file 3).

### Identification of aberrant SLC22A3 methylation by targeted bisulfite sequencing in MDS and AML patients

**Fig. 2:**
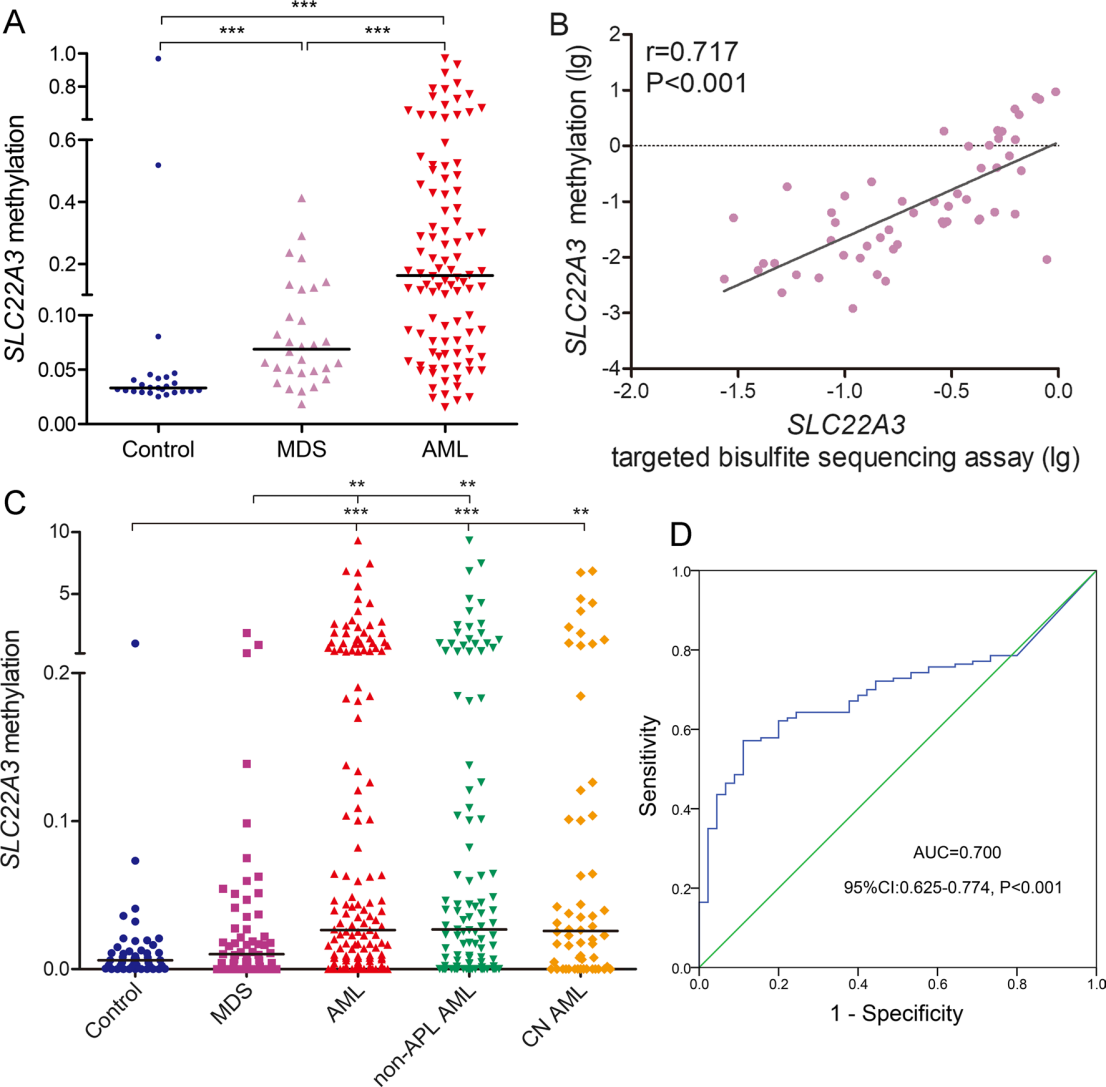
Confirmation of *SLC22A3* hypermethylation in AML. **A** *SLC22A3* methylation density in controls, MDS and AML patients detected by targeted bisulfite sequencing. **B** The correlation of *SLC22A3* methylation between two detection methods (RQ-MSP and targeted bisulfite sequencing). **C** *SLC22A3* methylation level in larger cohort of controls, MDS and AML patients analyzed by RQ-MSP. **D** ROC curve analysis by *SLC22A3* methylation for distinguishing AML patients from controls. **: *P* < 0.01; ***: *P* < 0.001

IN view of the significance of *SLC22A3* methylation and expression as mentioned above, we wanted to identify aberrant *SLC22A3* methylation involved in new diagnosed MDS and AML patients. We detected methylation pattern of CpG sites located at *SLC22A3* promoter region in 30 MDS, 100 AML patients and 25 controls using MethylTarget assay (targeted bisulfite sequencing). The mean bait coverage attached 1694 × , and Q30 was 75.56% [33]. The methylation level of *SLC22A3* was significantly increased in MDS and AML patients compared with controls (*P* < 0.001 and < 0.001), as well as in AML compared with MDS (*P* < 0.001; Fig. 2A).

### Confirmation of SLC22A3 hypermethylation in an expanded group of MDS and AML patients

To further confirm the pattern of *SLC22A3* methylation, we enrolled a larger cohort of newly diagnosed MDS (*n* = 61) and AML (*n* = 153) samples by RQ-MSP, a more convenient method. Primers for RQ-MSP and targeted sequencing were designed most overlapped in ampliconic sequence. In addition, RQ-MSP results showed a high positive correlation with that in the targeted bisulfite sequencing as confirmed (*r* = 0.717, *P* < 0.001, *n* = 54, Fig. 2B). As shown in Fig. 2C, *SLC22A3* methylation pattern was markedly higher in whole-cohort AML samples than in control and MDS groups (*P* < 0.001 and = 0.002), as well as in non-APL AML (*n* = 107) and CN AML (*n* = 58) vs. controls, respectively (*P* < 0.001 and = 0.002). However, we did not find a distinct variation between controls and MDS specimens.

### Clinical properties and genetic features of MDS and AML with high SLC22A3 methylation

According to ROC curve analysis, *SLC22A3* methylation could be helpful to distinguish AML from controls (Fig. 2D). To investigate the clinical correlation of *SLC22A3* methylation in pathogenesis and prognosis of MDS/AML, patients were divided into two distinct groups (*SLC22A3* hypermethylation and *SLC22A3* hypomethylation) based on the cutoff value of 0.042 (according to Youden index of ROC curve), which can also put almost all healthy donors in hypomethylation set. *SLC22A3* hypermethylation was associated with lower platelets and higher *CEBPA* mutation rate in non-APL AML patients (*P* = 0.020 and 0.006), as well as higher mutation rate of *N/K-RAS* in CN-AML (*P* = 0.080; Table 1). Besides, *SLC22A3* methylation pattern was irregularly distributed among leukemia subtypes (FAB) (Table 1). However, we observed no statistical differences between clinical data of two MDS groups (Table 2).

**Table 1:** Comparison of clinical manifestations and laboratory features between *SLC22A3* hypomethylated and hypermethylated AML patients

Patient's parameters	Non-APL AML			CN AML		
	Hypomethylated (*n* = 65)	Hypermethylated (*n* = 48)	*P* value	Hypomethylated (*n* = 37)	Hypermethylated (*n* = 22)	*P* value
Sex, male/female	38/27	31/17	0.562	22/15	12/10	0.789
Median age, years (range)	58 (18–85)	55 (18–85)	0.115	62 (18–85)	59 (18–79)	0.141
Median WBC, × 10^9^/L (range)	19.9 (0.9–528.0)	20.1 (0.4–165.8)	0.598	33.9 (1.2–528)	23.7 (0.9–135.4)	0.519
Median hemoglobin, g/L (range)	84 (32–133)	76 (34–144)	0.796	87 (32–123)	76 (47–144)	0.280
Median platelets, × 10^9^/L (range)	49 (9–447)	36 (5–264)	0.020	52 (9–234)	44 (7–148)	0.106
BM blasts, % (range)	50.8 (20.0–99.0)	60.5 (21.5–94.5)	0.276	58.5 (21.5–99)	58.0 (21.5–93)	0.714
FAB			0.254			0.535
M0	0 (0%)	2 (4.2%)		0 (0%)	1 (4.3%)	
M1	5 (7.7%)	4 (8.3%)		3 (7.9%)	1 (4.3%)	
M2	31 (47.7%)	24 (50%)		16 (42.1%)	9 (39.1%)	
M3	–	–		–	–	
M4	19 (29.2%)	7 (14.6%)		11 (28.9%)	4 (17.4%)	
M5	7 (10.8%)	9 (18.8%)		5 (13.2%)	6 (26.1%)	
M6	3 (4.6%)	2 (4.2%)		2 (5.3%)	1 (4.3%)	
Cytogenetic classification			0.616			
Favorable	5 (7.7%)	7 (14.6%)				
Intermediate	44 (67.7%)	31 (64.6%)				
Adverse	13 (20%)	9 (18.8%)				
No data	3 (4.6%)	1 (2.1%)				
Karyotype			0.715			
Normal	37 (56.9%)	22 (45.8%)				
t(8;21)	5 (7.7%)	6 (12.5%)				
t(16;16)	0 (0%)	1 (2.1%)				
t(v; 11q23)	2 (3.1%)	3 (6.3%)				
-5/5q-	0 (0%)	1 (2.1%)				
t(9;22)	1 (1.5%)	1 (2.1%)				
-7/7q-	3 (4.6%)	1 (2.1%)				
Complex	5 (7.7%)	6 (12.5%)				
Other	1 (1.5%)	1 (2.1%)				
No data	11 (16.9%)	6 (12.5%)				
Gene mutation						
CEBPA ( ±)	2/54	9/29	0.006	1/30	3/14	0.121
NPM1 ( ±)	8/49	6/32	1.000	7/25	5/12	0.729
FLT3-ITD ( ±)	4/52	3/35	1.000	4/27	2/15	1.000
c-KIT ( ±)	3/53	2/36	1.000	2/29	0/17	0.533
N/K-RAS ( ±)	4/52	7/31	0.113	2/29	5/12	0.080
IDH1/2 ( ±)	2/54	3/35	0.391	1/30	3/14	0.121
DNMT3A ( ±)	3/53	2/36	1.000	3/28	2/15	1.000
U2AF1 ( ±)	1/55	2/36	0.563	1/30	1/16	1.000
SRSF2 ( ±)	3/53	1/37	0.645	2/29	0/17	0.533
SETBP1 ( ±)	1/55	1/37	1.000	0/31	1/16	0.354

*non-APL AML* acute myeloid leukemia without FAB-M3, *CN-AML* cytogenetically normal AML, *WBC* white blood cells, *HB* hemoglobin, *PLT* platelet count, *BM* bone marrow, *FAB* French–American–British classification

**Table 2:** Comparison of clinical manifestations and laboratory features between SLC22A3 hypomethylated and hypermethylated MDS patients

Patient’s parameter	Hypomethylated (*n* = 38)	Hypermethylated (*n* = 10)	*P* value
Sex (male/female)	20/18	8/2	0.304
Age (years)	57 (27–83)	67 (28–84)	0.454
WBC (× 10^9^/L)	4.0 (1.2–82.4)	4.4 (1.2–19)	0.493
HB (g/L)	61 (35–140)	62 (46–107)	0.493
PLT (× 10^9^/L)	61 (0–1176)	56 (12–323)	0.919
BM blasts (%)	5 (0–19)	6 (0–17)	0.648
WHO classification			0.268
MDS-SLD/MLD	13	3	
MDS-RS	7	0	
MDS with isolated del(5q)	1	0	
MDS-EB1	5	4	
MDS-EB2	12	3	
Cytogenetic classification			1.000
Favorable	23 (60%)	7 (70%)	
Intermediate	6 (16%)	1 (10%)	
Adverse	6 (16%)	1 (10%)	
No data	3 (8%)	1 (10%)	
IPSS			0.476
Low	6 (16%)	0 (0%)	
Int-1	15 (39%)	7 (70%)	
Int-2	9 (24%)	1 (10%)	
High	5 (13%)	1 (10%)	
No data	3 (8%)	1 (10%)	
Gene mutations			
CEBPA ( ±)	0/31	0/10	
IDH1/2 ( ±)	1/30	0/10	1.000
DNMT3A ( ±)	0/31	0/10	
U2AF1 ( ±)	1/30	1/9	0.433
SF3B1 ( ±)	4/27	0/10	0.556
SRSF2 ( ±)	0/31	2/8	0.055
SETBP1 ( ±)	1/30	0/10	1.000

*WBC* white blood cells, *HB* hemoglobin, *PLT* platelet count, *BM* bone marrow, *IPSS* International Prognostic Scoring System, *WHO* World Health Organization, *MDS-SLD/MLD* MDS with signal lineage dysplasia/multilineage dysplasia, *MDS-RS* MDS with ringed sideroblasts, *MDS-EB* MDS with excess blasts, *IPSS* International Prognostic Scoring System

### Correlation of SLC22A3 hypermethylation with prognosis in MDS and AML patients

**Fig. 3:**
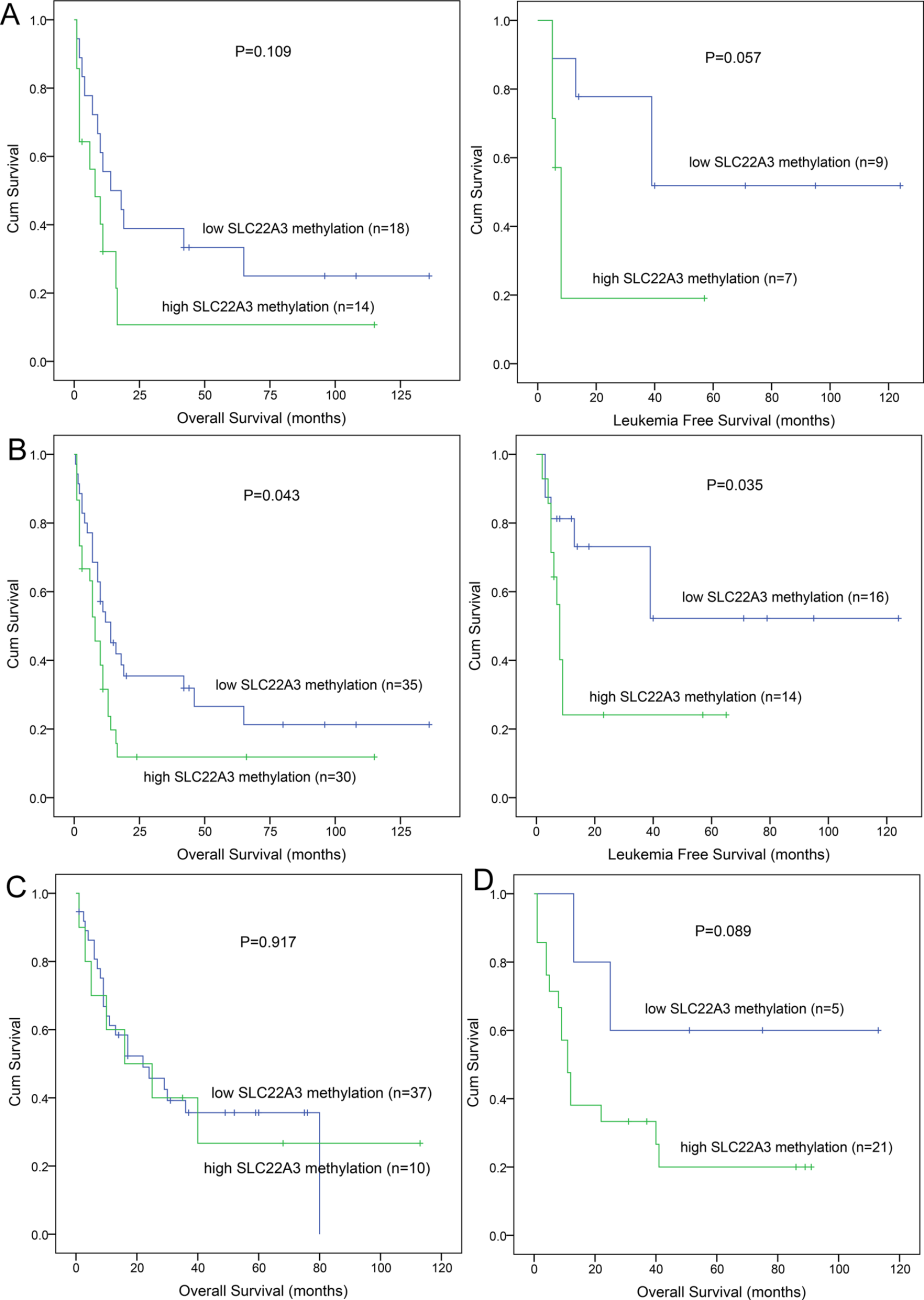
Prognostic value of *SLC22A3* methylation in AML and MDS patients. **A**, **B** The impact of *SLC22A3* methylation on OS and LFS of CN-AML, and non-APL AML patients, respectively. AML patients were treated by “3 + 7” induction regimens. **C**, **D** The impact of *SLC22A3* methylation on OS among MDS patients. SLC22A3 methylation was measured using RQ-MSP (**C**) and MethylTarget assay (**D**)

Based on follow-up investigation, we would like to analyze whether *SLC22A3* methylation affect the life span expectancy of MDS and AML patients. We screened AML patients treated with 3 days of an anthracycline and 7 days of cytarabine (“3 + 7” regimens). Kaplan–Meier analysis indicated tendencies of shorter OS and leukemia-free survival (LFS) in CN-AML patients with *SLC22A3* hypermethylation (*P* = 0.109 and 0.057; Fig. 3A). *SLC22A3* hypermethylation group showed conspicuous shorter OS and LFS in non-APL AML patients (*P* = 0.043 and 0.035; Fig. 3B). However, *SLC22A3* methylation pattern had no influence on MDS prognosis, no matter which method methylation data were measured (*P* > 0.050; Fig. 3C, D). Next, we enrolled variables that have statistically differences in univariate cox regression analysis (*P* < 0.200) and/or clinically recognized related to AML into multivariate analysis. In non-APL AML cohort, *SLC22A3* hypermethylation was an independent risk indicator for shorter OS and LFS, respectively (*P* = 0.001 and < 0.001; Table 3).

**Table 3:** Cox regression analyses of variables for survival in non-APL AML patients

	Univariate analysis		Multivariate analysis	
	HR (95% CI)	*P*	HR (95% CI)	*P*
*Overall survival*				
Age (> 60/ ≤ 60 years)	2.052 (1.322–3.184)	0.001	2.590 (1.621–4.137)	< 0.001
WBC(≥ 30/ < 30 × 10^9^/L)	1.921 (1.243–2.971)	0.003	1.426 (0.903–2.252)	0.128
PLT(≥ 100/ < 100 × 10^9^/L)	1.663 (0.956–2.890)	0.072	1.827 (1.037–3.219)	0.037
SLC22A3 methylation (high/low)	1.405 (0.905–2.180)	0.129	2.331 (1.432–3.794)	0.001
Cytogenetic classification	1.699 (1.214–2.378)	0.002	1.834 (1.291–2.606)	0.001
CEBPA mutation (±)	1.501 (0.682–3.303)	0.312	–	–
NPM1 mutation (±)	0.830 (0.379–1.820)	0.643	–	–
FLT3-ITD mutation (±)	0.822 (0.299–2.262)	0.705	–	–
DNMT3A mutation (±)	1.402 (0.507–3.872)	0.515	–	–
*Leukemia-free survival*				
Age (> 60/ ≤ 60 years)	2.001 (0.796–5.031)	0.140	7.158 (2.054–24.941)	0.002
WBC (≥ 30/ < 30 × 10^9^/L)	2.366 (0.977–5.727)	0.056	1.346 (0.508–3.570)	0.550
PLT (≥ 100/ < 100 × 10^9^/L)	4.340 (1.391–13.539)	0.011	19.235 (4.038–91.622)	< 0.001
SLC22A3 methylation (high/low)	2.062 (0.841–5.056)	0.114	19.856 (4.405–89.512)	< 0.001
Cytogenetic classification	2.836 (1.138–7.069)	0.025	11.686 (2.432–56.156)	0.002
CEBPA mutation (±)	1.587 (0.357–7.050)	0.544	–	–
NPM1 mutation (±)	0.665 (0.151–2.916)	0.588	–	–
FLT3-ITD mutation (±)	0.790 (0.104–5.991)	0.819	–	–
DNMT3A mutation (±)	1.760 (0.229–13.514)	0.587	–	–

### Transcriptional regulatory effects of SLC22A3 methylation on mRNA expression

**Fig. 4:**
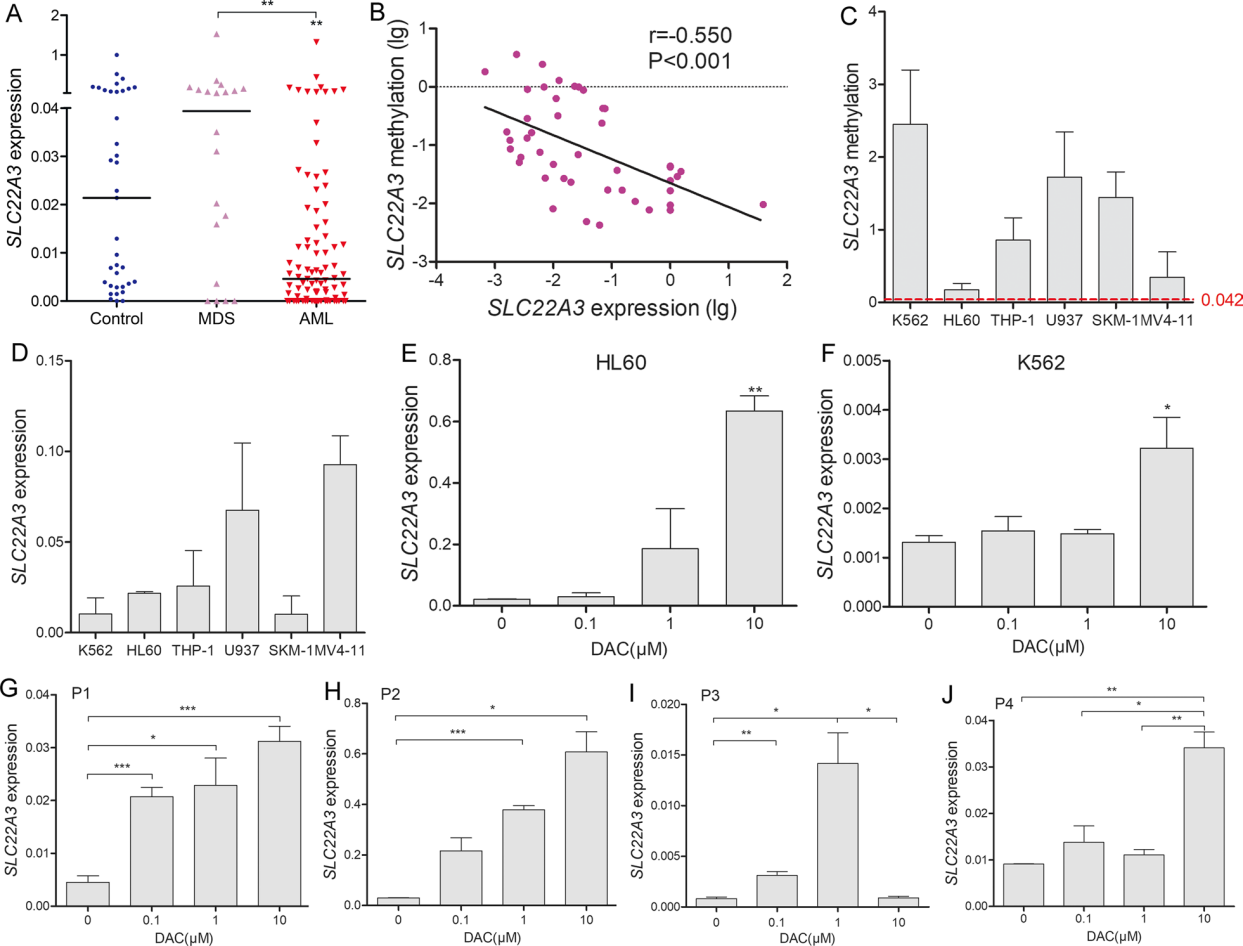
Regulation effect of DNA methylation on *SLC22A3* expression. **A** *SLC22A3* expression level detected by RT-qPCR in controls, MDS and AML patients. **B** Correlation between *SLC22A3* methylation (RQ-MSP) and expression (RT-qPCR). **C**, **D** *SLC22A3* methylation and expression pattern in six leukemic cell lines. **E**, **F** *SLC22A3* expression on HL60 and K562 changed with the concentration of DAC treatment (0, 0.1, 1, 10 μmol/L). **G**–**J** *SLC22A3* expression changed with the concentration of DAC treatment on AML fresh CMMNCs isolated from four newly diagnosed AML patients (Patient1-M2a, Patient2-M2b, Patient3-M4a, and Patient4-M5b). *: *P* < 0.05

To verify the regulatory role of *SLC22A3* methylation in AML pathogenesis, specimens of newly diagnosed MDS (*n* = 20) and AML (*n* = 89) were used to assess *SLC22A3* expression level by RT-qPCR. Our results showed that *SLC22A3* expression was downregulated in AML compared with controls and MDS (*P* = 0.001 and 0.002; Fig. 4A). Besides, we observed a credibly negative correlation between *SLC22A3* methylation and expression (*r* =  − 0.550, *P* < 0.001, *n* = 46, Fig. 4B).

DNA methylation and mRNA expression levels of *SLC22A3* were performed on six leukemia cell lines (Fig. 4C, D). Next we selected HL60 and K562 cells with the lowest and highest methylation levels, respectively, and treated them with the demethylation drug DAC. A dose–response relationship can be observed between DAC treatment and *SLC22A3* expression over a range of drug concentrations (Fig. 4E, F). Furthermore, we treated 4 AML fresh BMMNCs with graduate increased dosage of DAC, and found that DAC also promoted mRNA expression of *SLC22A3* in the same dose range (Fig. 4G–J), which further verify our thesis that DNA methylation regulates *SLC22A3* mRNA expression in AML.

### Assessment of SLC22A3 expression for AML prognosis as a surveillance biomarker

**Fig. 5:**
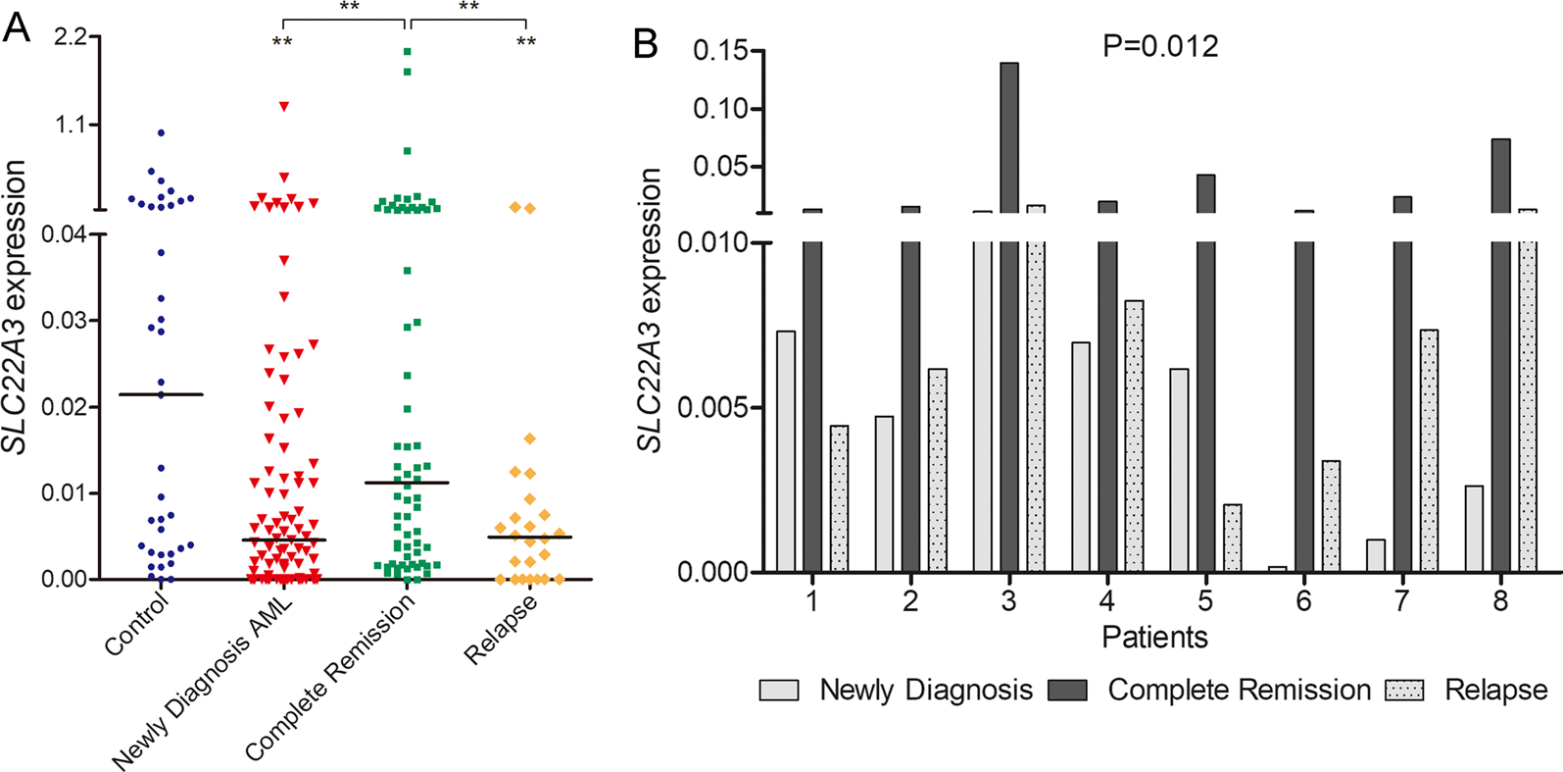
*SLC22A3* expression in the surveillance of AML. **A** *SLC22A3* expression in different clinical stages (new diagnosis, complete remission, and relapsed time) of AML patients. **B** Dynamic change of *SLC22A3* expression in eight patients during different clinical stages

Since *SLC22A3* methylation level is associated with prognosis of AML, we attempted to further evaluate whether *SLC22A3* expression level reflects the course of AML disease. We collected AML specimens from different clinical stages including 66 patients who achieved CR after induction therapy and 24 relapsed patients. RT-qPCR results revealed that *SLC22A3* expression level was significantly improved in CR patients compared to newly diagnosed time, and subsequently fell back in the relapse phase (*P* = 0.002 and 0.009; Fig. 5A). As presented in Fig. 5B, *SLC22A3* expression exhibited obviously dynamic changes with the clinical phases in a follow-up study of eight patients (*P* = 0.012).

### Biological function of SLC22A3 on leukemia cells

**Fig. 6:**
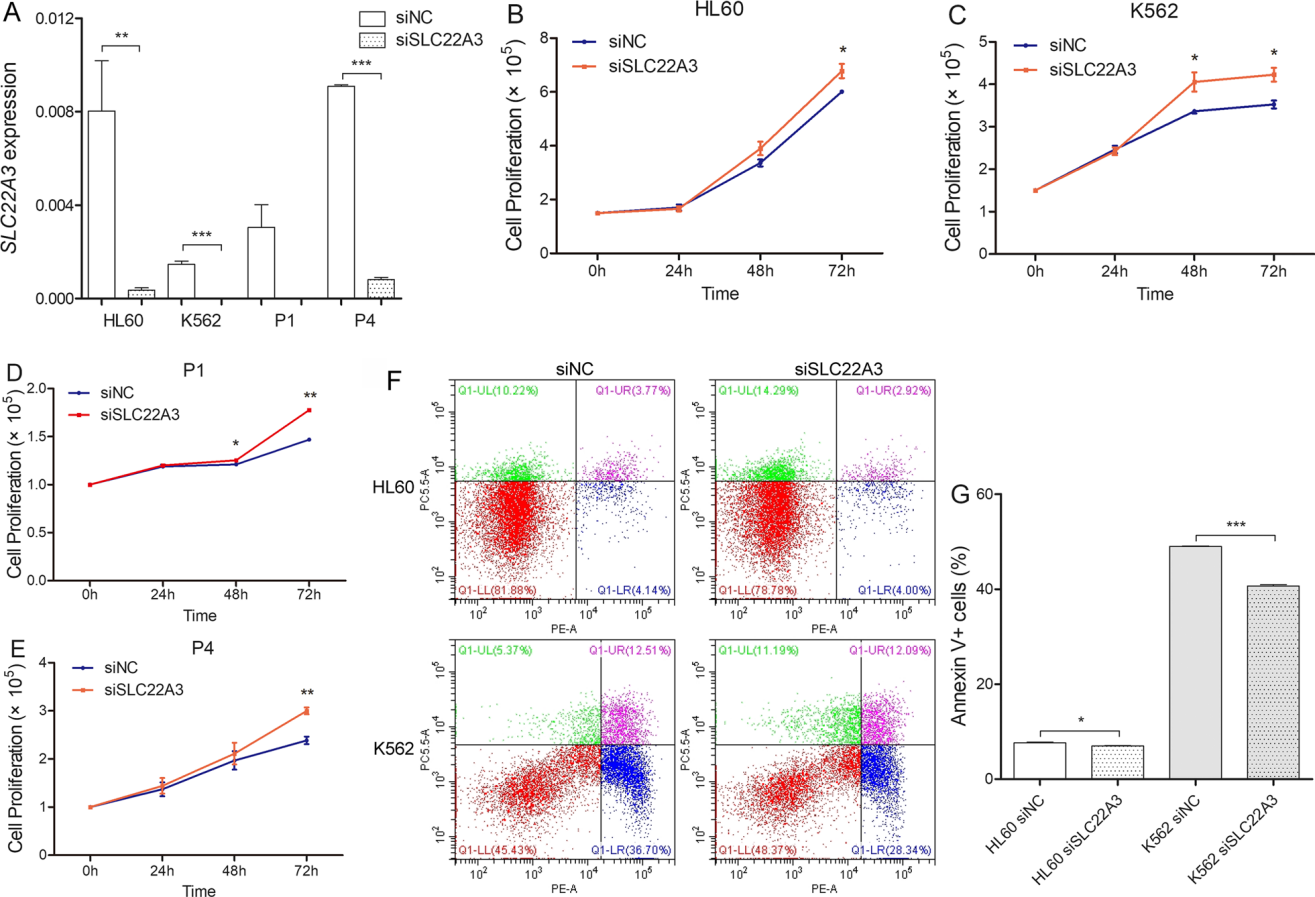
Biological role of *SLC22A3* on leukemic cell lines and AML fresh BMMNCs. **A** Transcript level of *SLC22A3* in HL60, K562 and 2 AML fresh BMMNCs (patient1 and patient4) after transfected with small interfering (si)RNA against *SLC22A3* and negative control. **B**–**E** The effect of *SLC22A3* under-expression on cell proliferation. *SLC22A3* under-expression significantly increased cell proliferation ability in HL60, K562, and fresh BMMNCs from Patient1 and Patient4. **F**, **G** The effect of *SLC22A3* under-expression on cell apoptosis. *SLC22A3* under-expression significantly decreased cell apoptosis ratio in HL60 and K562 cells

TO explore the biological role of *SLC22A3* expression in AML, we created conditions of *SLC22A3* silencing in HL60 and K562 cells, as well as fresh BMMNCs from two AML patients. We transfected siSLC22A3 and its related siNC into these tool cells, and obtained remarkable silencing effect (Fig. 6A). With the knock-down of *SLC22A3* transcript level, proliferation ability of HL60, K562, and AML fresh BMMNCS were significantly climbing among 72 h (Fig. 6B–E). Compared with siNC controls, in addition, HL60 siSLC22A3 and K562 siSLC22A3 exhibited lesser apoptosis after culturing in starvation station for 72 h (Fig. 6F, G). In addition, pro-apoptotic effect of DAC was weakened in K562 siSLC22A3 after treated for 24 h, compared with siNC (20.24% and 65.63%, Additional file 4).

### Molecular exploration of SLC22A3 in AML

**Fig. 7:**
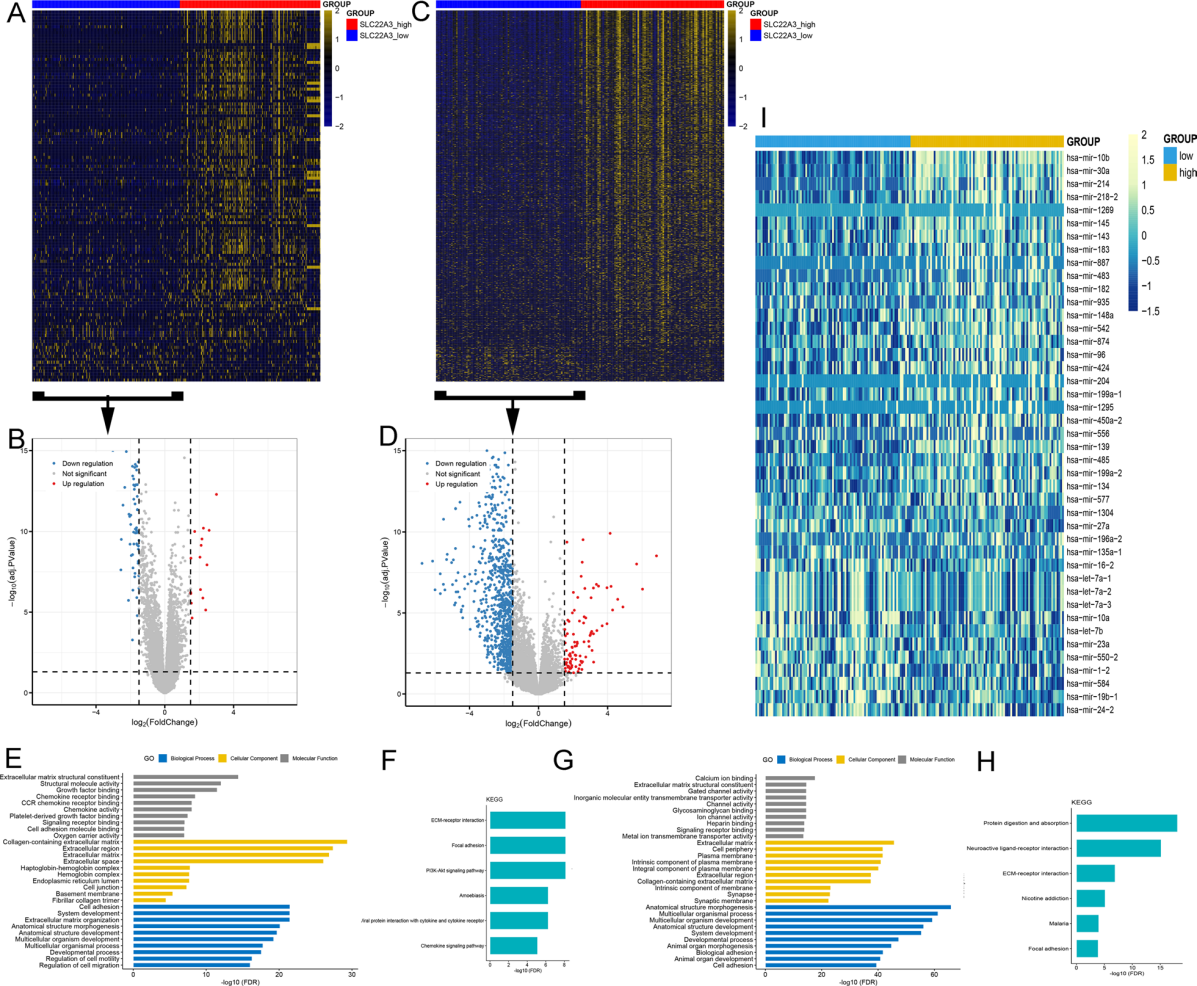
Molecular signatures associated with *SLC22A3* in AML. **A** Heatmap of differentially expressed genes between patients with low- and high-expressed *SLC22A3* among Beat AML datasets (FDR < 0.05, *P* < 0.05, and |log2 FC|> 1.5). **B** Volcano plot of up- and downregulated genes in lower expressed *SLC22A3* group among Beat AML datasets (FDR < 0.05, *P* < 0.05, and |log2 FC|> 1.5). **C** Heatmap of differentially expressed genes between AML patients with low- and high-expressed *SLC22A3* among TCGA datasets (FDR < 0.05, *P* < 0.05, and |log2 FC|> 1.5). **D** Volcano plot of up- and downregulated genes in lower expressed *SLC22A3* group among TCGA datasets (FDR < 0.05, *P* < 0.05, and |log2 FC|> 1.5). **E**, **F** Gene ontology (GO) and Kyoto Encyclopedia of Genes and Genomes (KEGG) analysis of differentially expressed genes among Beat AML datasets by using online website of STRING (http://string-db.org). **G**, **H** GO and KEGG analysis of differentially expressed genes among TCGA datasets by using online website of STRING. **I** Heatmap of differentially expressed microRNAs between AML patients with low- and high-expressed *SLC22A3* among TCGA datasets (FDR < 0.05, *P* < 0.05, and |log2 FC|> 1.5)

To further understand the biological insight of *SLC22A3* in AML, we analyzed the transcriptome differences to be associated with *SLC22A3* expression from Beat AML and TCGA cohorts. AML patients were divided by the median level of *SLC22A3* expression into low- and high-expression groups. We identified 124 differentially expressed genes in Beat AML (Fig. 7A, B; Additional file 5) and 1015 in TCGA (Fig. 7C, D; Additional file 6). Combined with the results of the above two cohorts, a total of 66 positively correlated genes were singled out, including VCAM1, SOX9, ID4, and ITIH5 which have been clearly reported for antileukemia effects [34–37]. Moreover, these genes were concentrated in the extracellular matrix and involved in signaling receptor binding, cell adhesion, and ECM-receptor interaction according to Gene Ontology (GO) and Kyoto Encyclopedia of Genes and Genomes (KEGG) enrichment analysis (Fig. 7E–H). Besides, the differentially expressed analysis based on microRNA data of TCGA screened out 43 microRNAs including 16 negatively associated with *SLC22A3* (Fig. 7I; Additional file 7). These negatively correlated microRNAs such as hsa-let-7b, hsa-mir-19b, and hsa-mir-196a were involved in antileukemia effects as previously reported [38–40].

## Discussion

Hematopoietic malignancies are more susceptible to epigenetic interventions than solid malignancies. AML is a heterogeneous myeloid tumor that displays extensive variation in their clinical courses and in response to therapy. Epigenetics allows us to extend the exploration of the potential diversity among AML subsets [41]. In this research, we have found that *SLC22A3* present general higher methylation and lower expression pattern in the whole-cohort de novo AML, non-APL AML and CN patients. Set a threshold for *SLC22A3* methylation that can exclude almost all healthy donors, AML patients above this level tended to have lower OS and LFS. The similar poor prognosis tendencies have also been reflected in *SLC22A3* under-expression groups among large AML samples from TCGA and OHSU database. Follow-up study and paired analysis provided a more intuitive view in dynamic changes of *SLC22A3* expression with the clinical phases, which can promote its understanding and further application in AML surveillance. The genomes hypomethylation and aberrant hypermethylation in promoter are involved in many kinds of tumorigenesis, leading to activating of proto-oncogenes and inhibiting of tumor suppressor genes [42, 43]. Abnormal DNA methylation and expression levels of *SLC22A3* have been demonstrated in several tumors. Higher methylation and lower expression of *SLC22A1* and *SLC22A3* was observed in hepatocellular carcinoma and prostate tumor compared with matched normal samples [44, 45]. SLC22A1 activity was reported to correlate with the sensitivity of imatinib, a tyrosine kinase inhibitor, in patients with chronic myeloid leukemia [46, 47]. In this study, our results based on clinical BM specimens and bioinformatics analysis reveal an association between *SLC22A3*-hypermethyl status and AML.

Our study showed that the DNA methylation level of *SLC22A3* was also increased in MDS group compared with the control group, but lower than that of AML. MDS is a group of malignant clonal diseases with a high risk of transition to AML. However, MDS is a highly heterogeneous group of diseases, whose clinical course and outcome vary greatly, with no more than 30% actually transforming into AML [48, 49]. Overall, MDS and AML represent a disease continuum that undergoes genetic clonal evolution, but there are still differences in pathophysiology between AML and MDS.

The transcription factor CCAAT enhancer binding protein alpha (CEBPA) is a myeloid transcription factor. Ley et al. confirmed that mutations in CEBPA and other myeloid transcription factor genes, such as DNMT3A, NPM1, IDH1/2 and RUNX1, were common in AML and suggested that these mutations had functions related to the pathogenesis of AML [31]. Results of our clinical analysis showed that *SLC22A3* DNA hypermethylation status was associated with CEBPA mutation in AML patients. However, we did not discuss two types (biallelic and single heterozygous) of CEBPA mutations separately because of the limited mutation cases. We observed no correlation between SLC22A3 expression and CEBPA biallelic mutations based on Beat AML databases. Considering of the prognosis differences between two types of CEBPA mutations, the causal relationship between *SLC22A3* hypermethylation and CEBPA mutation and its clinical significance in AML remain to be clarified.

Promoter methylation plays an important role in SLC22A3 expression. Chen et al. confirmed that methylation in the SLC22A3 promoter region could explain the low expression level of SLC22A3 in high-Gleason grade prostate cancer, which may be related to the progression of prostate cancer. In SLC22A3-negative HCT116, SLC22A3 mRNA levels were significantly reactivated with increased dose of 5′ -AZADC (a demethylating agent) [44]. Xiong et al. observed that *SLC22A3* methylation conferred susceptibility to esophageal squamous cell carcinoma [50].

Our demethylation studies with DAC also showed that *SLC22A3* mRNA expression of two leukemic cell lines and AML bone marrow mononuclear cells increased with increasing drug dose within a certain concentration range. The SLC22A3 post-intervention experiments confirmed that SLC22A3 downregulation led to active proliferation and diminished apoptosis of leukemia cells. SLC22A3 is a widely expressed drug transporter. hSLC22A3-mediated oxaliplatin uptake in cancer is thought to be important for its cytotoxicity [16], but it is not clear whether SLC22A3 mediated the uptake of DAC. Our results showed that DNA hypermethylation may repress drug importer SLC22A3 located in membrane and resulted in enhanced drug resistance and diminished apoptosis. We considered that poor clinical outcomes of SLC22A3 downregulation/SLC22A3 hypermethylation patients could be associated with weakened drug pump function.

In summary, this current study showed that *SLC22A3* DNA is aberrant hypermethylation in AML and different clinical status of disease display distinct patterns of DNA methylation. DNA methylation levels may be useful for AML prognosis. Just as Šestáková et al. [51] proposed, further validation of selected tumor markers is important especially for their clinical applications. Future studies are needed to investigate more cases to clarify the significance of *SLC22A3* methylation level and expression in therapy of AML and to clarify whether *SLC22A3* aberrant methylation facilitate or merely coexist with *CEBPA* mutation.

## Conclusion

Our results showed that increased methylation and decreased expression of SLC22A3 may be indicators of poor prognosis in AML. Methylation-silenced SLC22A3 expression may have potential guiding significance on the antileukemia effect of DAC.

## Supplementary Information

**Additional file 1**: Identification of aberrantly hypermethylated SLCs in AML. **A** Heatmap of differentially methylated genes between normal and AML bone marrow specimens from GSE63409. **B** The Venn diagram of hypermethylated genes in AML. The intersection of hypermethylated genes in AML based on GSE63409 and the RRBS data that our lab has submitted to NCBI SRA databases previously (accession number PRJNA670308). **C** represents normal donors.

**Additional file 2:** Correlation between DNA methylation and mRNA expression of SLCs in AML from TCGA database. A-D, SLC5A8, SLC6A11, SLC7A14, SLC34A2. The values of zero were excluded from log calculation.

**Additional file 3**: SLC22A3 expression in genetics subsets of Beat AML cohort. A, SLC22A3 expression in subsets of recurrent genetic abnormalities. B, SLC22A3 expression in subsets of MLL rearranged AML. C-E, SLC22A3 expression in AML with CEBPA biallelic, NPM1, and FLT3-ITD mutations.

**Additional file 4: **Apoptotic analysis of K562 siNC/siSLC22A3 treated by DAC. **A**, **B**, Comparison of cell apoptosis between K562 siSLC22A3 and siNC after DAC dosing.

**Additional file 5: **Differently expressed mRNAs between lower and higher SLC22A3 expression groups from beat AML.

**Additional file 6: **Differently expressed mRNAs between lower and higher SLC22A3 expression AML groups from TCGA.

**Additional file 7:** Differently expressed microRNAs between lower and higher SLC22A3 expression AML groups from TCGA.

## Data Availability

The datasets used and/or analyzed during the current study are available from the corresponding author upon reasonable request.

## Abbreviations

AML: Acute myeloid leukemia
RRBS: Reduced representation bisulfite sequencing
BMMNCs: Bone marrow mononuclear cells
DAC: 5-Aza-2′-deoxycytidine
ATRA: All transretinoic acid
MDS: Myelodysplastic syndrome
CR: Complete remission
FAB: French–American–British
WHO: World Health Organization
IPSS: International Prognostic Scoring System
PQ-MSP: Real-time quantitative methylation-specific PCR
RT-qPCR: Real-time quantitative PCR
TCGA: The Cancer Genome Atlas
AUC: Area under the ROC curve
DFS: Disease-free survival
LFS: Leukemia-free survival
OS: Overall survival
APL: Acute promyelocytic leukemia
CN-AML: Cytogenetically normal AML
GO: Gene Ontology
KEGG: Kyoto Encyclopedia of Genes and Genomes
