# Supplementary figures and images for: SLC22A3 methylation-mediated gene silencing predicts adverse prognosis in acute myeloid leukemia
Source: Clin Epigenetics. 2022 Dec 2;14:162. doi: 10.1186/s13148-022-01373-w (PMC9716704; doi:10.1186/s13148-022-01373-w)

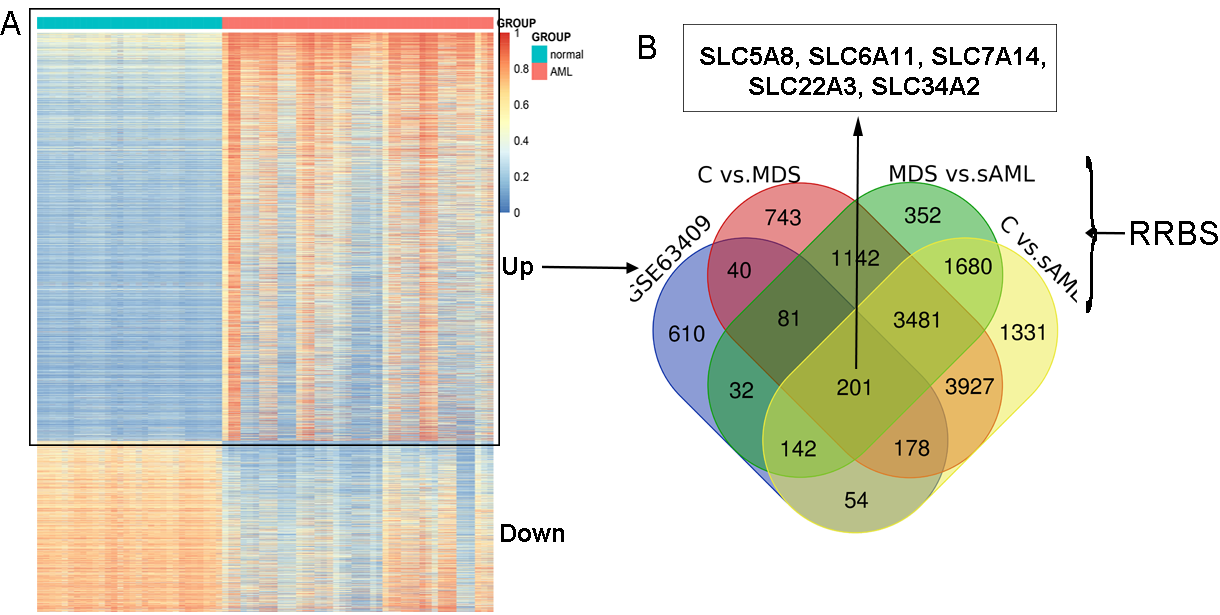

Supplement: Supplementary file 1 — Additional file 1: Identification of aberrantly hypermethylated SLCs in AML. A Heatmap of differentially methylated genes between normal and AML bone marrow specimens from GSE63409. B The Venn diagram of hypermethylated genes in AML. The intersection of hypermethylated genes in AML based on GSE63409 and the RRBS data that our lab has submitted to NCBI SRA databases previously (accession number PRJNA670308). C represents normal donors. [file 13148_2022_1373_MOESM1_ESM.tif]

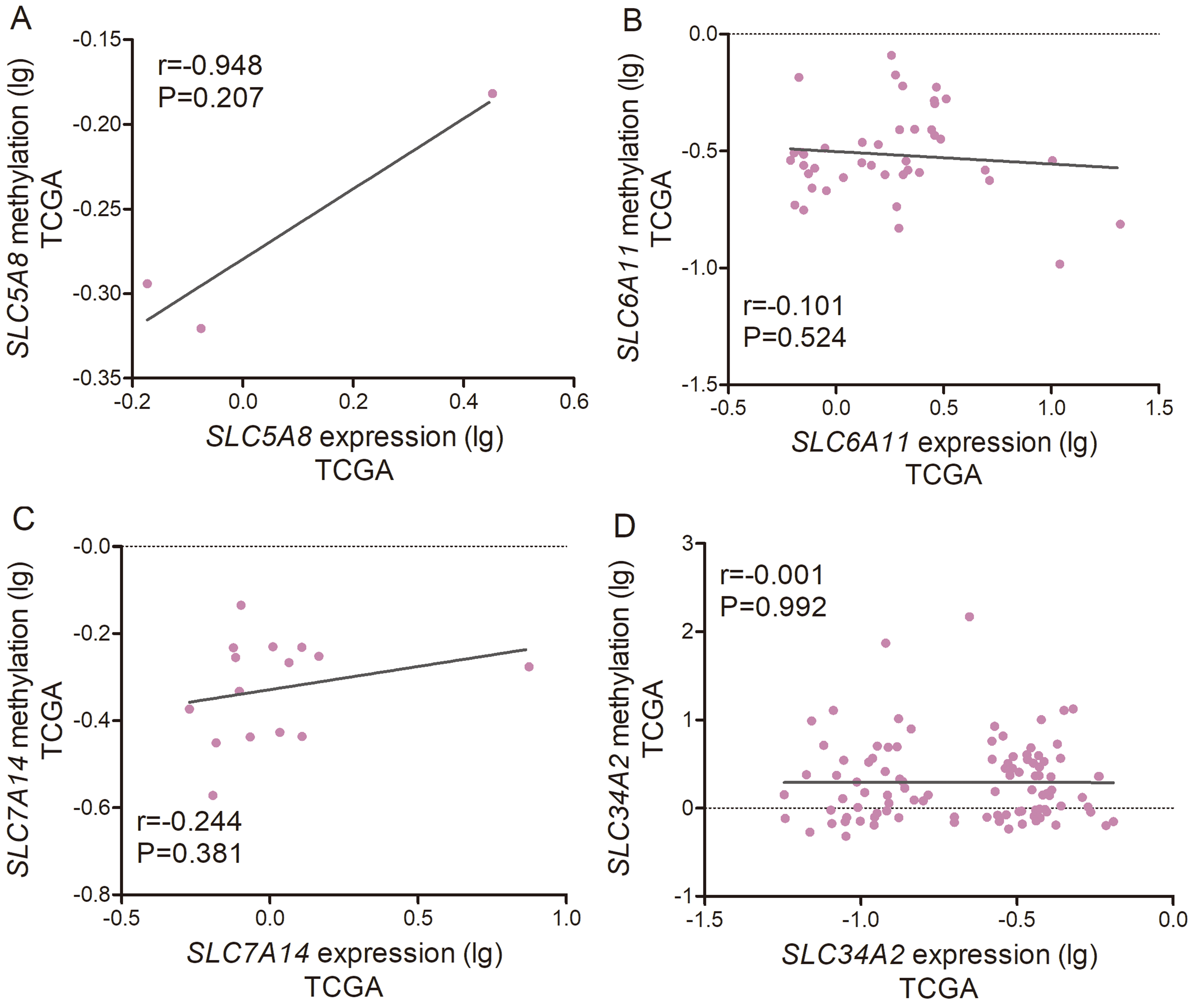

Supplement: Supplementary file 2 — Additional file 2: Correlation between DNA methylation and mRNA expression of SLCs in AML from TCGA database. A-D, SLC5A8, SLC6A11, SLC7A14, SLC34A2. The values of zero were excluded from log calculation. [file 13148_2022_1373_MOESM2_ESM.tif]

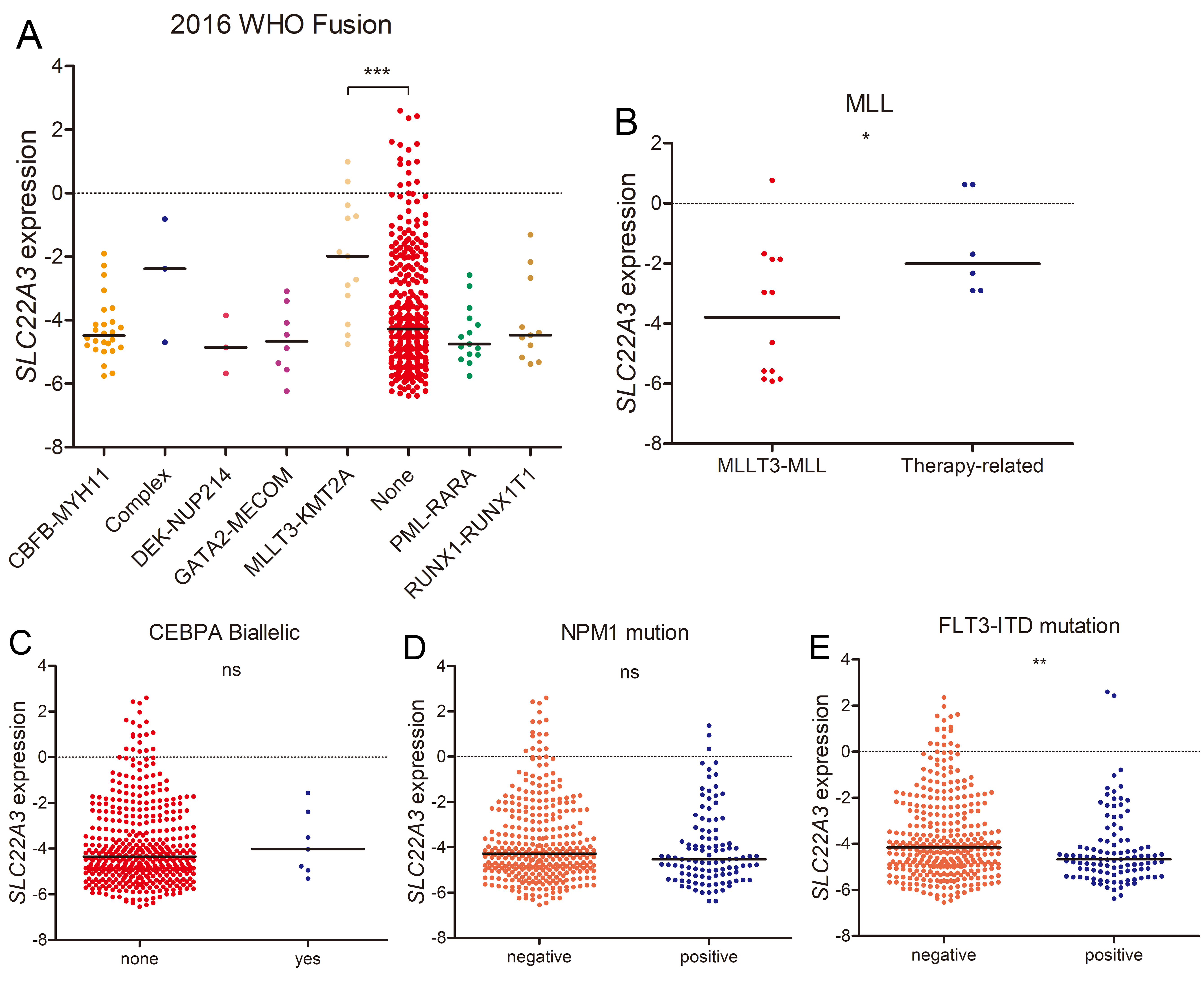

Supplement: Supplementary file 3 — Additional file 3: SLC22A3 expression in genetics subsets of Beat AML cohort. A, SLC22A3 expression in subsets of recurrent genetic abnormalities. B, SLC22A3 expression in subsets of MLL rearranged AML. C-E, SLC22A3 expression in AML with CEBPA biallelic, NPM1, and FLT3-ITD mutations. [file 13148_2022_1373_MOESM3_ESM.tif]

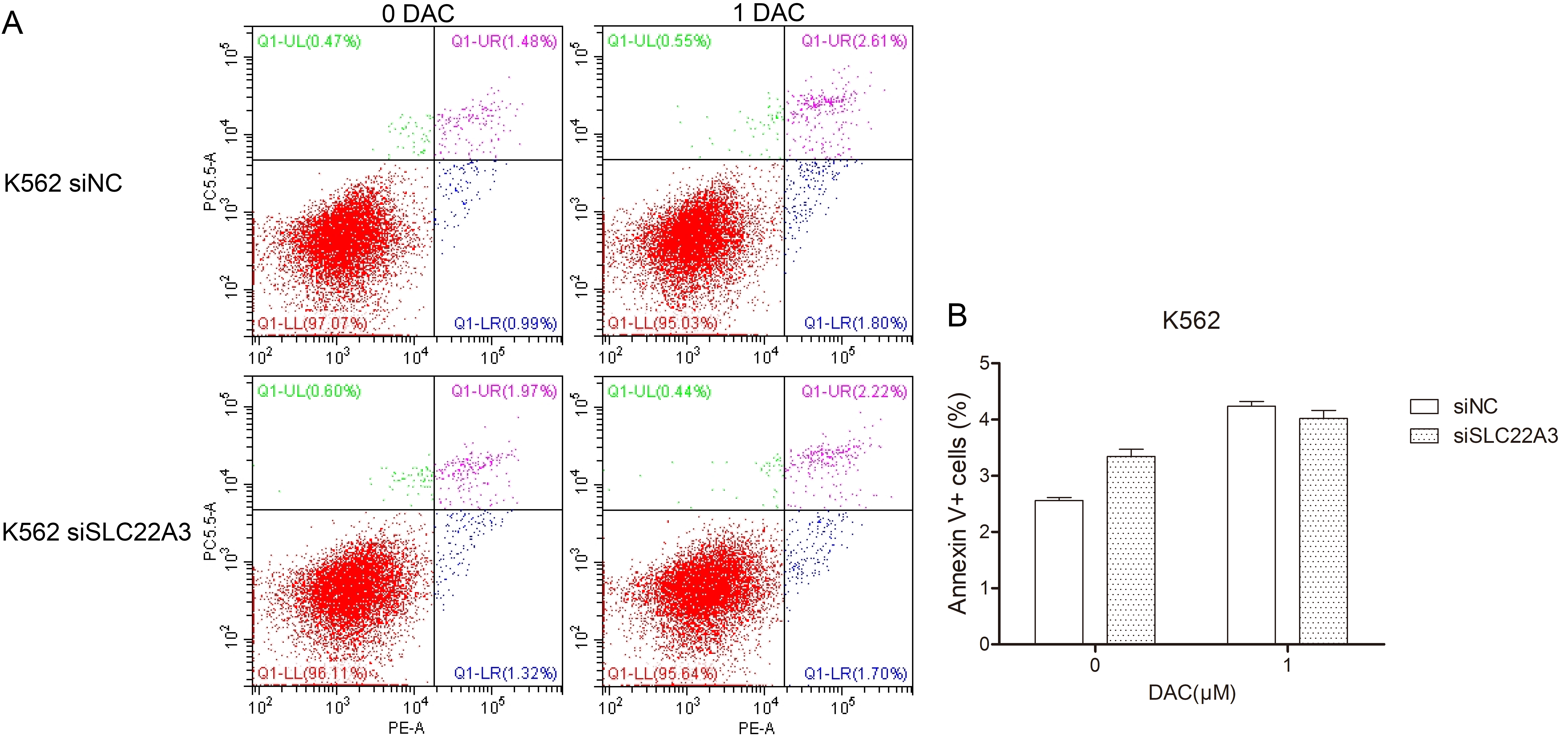

Supplement: Supplementary file 4 — Additional file 4: Apoptotic analysis of K562 siNC/siSLC22A3 treated by DAC. A, B, Comparison of cell apoptosis between K562 siSLC22A3 and siNC after DAC dosing. [file 13148_2022_1373_MOESM4_ESM.tif]
